# Chemotherapy induces adaptive drug resistance and metastatic potentials via phenotypic CXCR4-expressing cell state transition in ovarian cancer

**DOI:** 10.1371/journal.pone.0171044

**Published:** 2017-02-14

**Authors:** Hyun Hee Lee, Vanessa Bellat, Benedict Law

**Affiliations:** Molecular Imaging Innovations Institute, Department of Radiology, Weill Cornell Medicine, New York, New York, United States of America; University of South Alabama Mitchell Cancer Institute, UNITED STATES

## Abstract

Ovarian cancer (OVC) patients who receive chemotherapy often acquire drug resistance within one year. This can lead to tumor reoccurrence and metastasis, the major causes of mortality. We report a transient increase of a small distinctive CXCR4^High^/CD24^Low^ cancer stem cell population (CXCR4^High^) in A2780 and SKOV-3 OVC cell lines in response to cisplatin, doxorubicin, and paclitaxel, treatments. The withdrawal of the drug challenges reversed this cell-state transition. CXCR4^High^ exhibits dormancy in drug resistance and mesenchymal-like invasion, migration, colonization, and tumor formation properties. The removal of this cell population from a doxorubicin-resistant A2780 lineage (A2780/ADR) recovered the sensitivity to drug treatments. A cytotoxic peptide (CXCR4-KLA) that can selectively target cell-surface CXCR4 receptor was further synthesized to investigate the therapeutic merits of targeting CXCR4^High^. This peptide was more potent than the conventional CXCR4 antagonists (AMD3100 and CTCE-9908) in eradicating the cancer stem cells. When used together with cytotoxic agents such as doxorubicin and cisplatin, the combined drug-peptide regimens exhibited a synergistic cell-killing effect on A2780, A2780/ADR, and SKOV-3. Our data suggested that chemotherapy could establish drug-resistant and tumor-initiating properties of OVC via reversible CXCR4 cell state transition. Therapeutic strategies designed to eradicate rather than antagonize CXCR4^High^ might offer a far-reaching potential as supportive chemotherapy.

## Introduction

Epithelial ovarian cancer (EOC) patients who receive chemotherapy often develop drug resistance within one year. This often leads to a disease recurrence rate as high as 80%, accompanied by uncontrolled trascoelomic and hematogenous metastases. A tumor is composed of heterogeneous cell populations. Conventional chemotherapy can eradicate most cancer cells, but leaves small populations of innately drug-resistant cancer stem cells (CSCs) behind. These residual CSCs, with metastatic potentials, can remodel tumors to become more drug resistant.

Stromal-derived factor (SDF-1 or CXCL12), a CXC chemokine, and its cognate seven-transmembrane G-protein coupled receptor 4 (CXCR4) play significant roles in promoting tumorigenesis and drug resistance [1–6]. In ovarian cancer (OVC) cells, the SDF-1/CXCR4 axis is mediated by the vascular endothelial cell growth factor, endolyn, Notch pathway, and nuclear factor of activated T-cells 3 transcription factor [7–10]. The binding of SDF-1 to CXCR4 induces αvβ6 integrin and upregulates certain tumor-associated proteases including urokinase plasminogen activator and metalloproteinase 9 to promote cancer invasion [11, 12]. High levels of SDF-1 and CXCR4 in malignant ascites [10], and in the tumors and sera of OVC patients correlate to drug resistance, poor disease prognosis, and metastasis [13–16]. All the aforementioned studies suggest that CXCR4 can be an important target for OVC treatment.

In the past, therapeutic strategies were proposed to target the SDF-1/CXCR4 axis of different cancers [17–19]. Particularly in OVC, silencing CXCR4 expression by siRNA reduces tumor growth [20]. AMD3100 is a small molecule antagonist of CXCR4 that has been shown to promote the apoptosis of tumor cells and the local T-cell mediated immune responses [21]. A dimerized CXCR4-binding peptide, CTCE-9908, was reported to induce abnormal mitosis [22]. Using antibodies to neutralize SDF-1/CXCR4 could sensitize hyperthermia treatment [23]. However, all these approaches only exhibit modest anticancer effects, presumably because the drugs only block the activities of SDF-1/CXCR4 axis instead of eliminating the cancer cells. In fact, a majority of CXCR4 receptors are stored intracellularly [10, 24, 25]. The exposure of the cell-surface CXCR4 is a hallmark of a small distinctive cancer stem cell (CSC) that displays tumor formation, invasion, and migration capabilities accompanied with an innate resistance to chemotherapy [26]. Understanding the functional roles of this cell population would allow for the design of effective strategies to encounter drug resistance and metastasis, the common causes of mortality in OVC patients. In this study, we investigated how chemotherapy transiently enriches a small CXCR4^High^/CD24^Low^ cancer stem cell population (CXCR4^High^) to acquire adaptive drug resistance. This CXCR4^High^ displayed a mesenchymal nature: enhanced migration, invasion, and colony formation properties. We used a CXCR4-targeting peptide drug (CXCR4-KLA) comprised of a CXCR4 binding sequence (KPVSLSYRC) conjugated to a mitochondrial disruption domain (KLA) to demonstrate the therapeutic merits of eradicating CXCR4^High^. This peptide exhibited preferential cytotoxicity toward CXCR4^High^ and could be used with other cytotoxic agents to achieve synergistic OVC cell-killing effect.

## Materials and methods

### Cell culture

A2780 was purchased from Sigma (St. Louis, MO). Its doxorubicin-resistant cell lineage, A2780/ADR, was generated with weekly treatment of doxorubicin (100 ng/mL) to maintain consistent drug resistance. Both cell lines were maintained in RPMI 1640 media supplemented with 10% (w/v) FBS (HyClone, South Logan, UT) and 1% (w/v) antibiotics (penicillin-streptomycin) at 37°C in a humidified atmosphere of 5% CO_2_. SKOV-3 was purchased from ATCC (Manassas, VA) and the GFP-luciferase transfected SKOV-3 (SKOV-3/GFP-Luc) was purchased from Cell Biolabs (San Diego, CA). Both cell lines were maintained in McCoy’s 5A media supplemented with 10% (w/v) FBS (HyClone, South Logan, UT) and 1% antibiotics (penicillin-streptomycin) at 37°C in a humidified atmosphere of 5% CO_2_.

### Synthesis of CXCR4-KLA peptide

CXCR4-KLA, (KPVSLSYRCGGKLAKLAKKLAKLAK), and its FITC-labeled analogue were synthesized on a PS3 peptide synthesizer (Protein Technologies, Tucson, AZ), employing the Fmoc strategy on rink amide [27]. The peptide was purified by reverse-phase HPLC to >98% purity and the expected molecular weights were confirmed by MALD-TOF mass spectrometer.

### Quantitative real-time PCR

Quantitative real-time PCR was performed using the StepOnePlus^™^ Real-Time PCR System by monitoring the real-time increase in fluorescence of the SYBR Green dye (Life Technologies Corp., Grand Island, NY). Total RNAs were isolated using the TRIzol reagent (Life Technologies Corp., Grand Island, NY) and subsequently reverse transcribed to the cDNA using a SuperScript First-Strand Synthesis System (Invitrogen, Grand Island, NY). The quantitative RT-PCR was performed using a SYBR^®^ Green PCR Master mix (Applied Biosystems, Grand Island, NY) according to the manufacturer’s instructions. The reaction mix was subjected to real-time PCR to detect levels of the corresponding PCR primers. The specificity of each primer pair was confirmed by melting curve analysis. The employed PCR primer pairs are shown in S1 Table.

### Western blot

The total cell extracts were obtained using a Cell Lysis Buffer (Cell Signaling Technology Inc., Danvers, MA) in the presence of protease inhibitor (Roche Applied Science, Indianapolis, IN) at 4°C for 30 min. Briefly, cells (5x10^6^) were washed twice with cell wash solution. The extracted proteins (40 μg) was resolved by SDS-PAGE, transferred to nitrocellulose membranes using the Novex^™^ Bolt^™^ gel electrophoresis system and iBlot^®^ transfer stack system (Life Technologies Corp., Grand Island, NY), and probed with the appropriate dilution of CXCR4 antibody (1:1000, ab2074, Abcam, Cambridge, MA), β-actin antibody (1:10000, A5441, Sigma, St. Louis, MO) Na, K-ATPase antibody (1:1000, #3010, Cell Signaling Technology Inc., Danvers, MA), E-Cadherin (1:1000, #3195, Cell Signaling, Danvers, MA), and Vimentin antibody (1:1000, #3932, Cell Signaling Technology Inc., Danvers, MA). Immunoreactivity was detected using an ECL detection system (GE Healthcare Bio-Sciences Corp., Pittsburgh, PA). Films were exposed at multiple time points to ensure that images were not saturated.

### FACS analysis and cell sorting

The cells were detached with Accutase^®^ solution (Sigma, St Louis, MO), washed, re-suspended in ice-cold PBS, and then incubated for 30 min at 4°C with the following anti-human fluorophore-labeled monoclonal antibodies: CD44-APC (clone BJ18, BioLegend, San Diego, CA), CD133-APC (clone AC133, Miltenyi Biotec Inc., San Diego, CA), CXCR4-APC (clone 12G5, BioLegend, San Diego, CA), and CD24-FITC (clone ML5, BioLegend, San Diego, CA). Cells (5x10^3^/sample) were counted using a FACS (Beckman Coulter Gallios^™^, Indianapolis, IN). The data were analyzed with Kaluza analysis software. For specific binding analysis, cells (2x10^6^) were incubated with CXCR4-KLA-FITC (10 μM) for 2 h at 37°C in 1.5% (w/v) FBS-containing medium. Subsequently, cells were detached, washed, re-suspended in ice-cold PBS, and FACS analysis was performed to determine the binding of a FITC-labeled CXCR4-KLA to CXCR4^Low^ and CXCR4^High^. Cells (2x10^4^/sample) were counted using a FACS (Beckman Coulter Gallios^™^, Indianapolis, IN) and the data were analyzed with Kaluza analysis software.

Cell sorting was performed using FACSVantage (BD Bioscience, CA). Cells (2x10^7^) were stained with CXCR4-APC (clone 12G5, BioLegend, San Diego, CA) for 1 h at 4°C under sterile conditions. The isolated CXCR4^Low^ and CXCR4^High^ subpopulations were further characterized for purity by FACS analysis, as described above.

### Proliferation assay

Cells (5x10^3^) were seeded on a 96-well clear bottom plate. After 24 h, cells were incubated with fresh media containing indicated concentrations and combination treatment concentrations of doxorubicin (LC Laboratories, Woburn, MA), cisplatin (Sigma, St Louis, MO), paclitaxel (LC Laboratories, Woburn, MA), fluorouracil (Sigma, St Louis, MO), AMD3100 (Sigma, St Louis, MO), CTCE 9908 (Tocris Bioscience, Bristol, UK), and CXCR4-KLA. At the indicated times, CellTiter 96^®^ AqueousOne Solution Reagent (Promega, Madison, WI) was added to each well according to the manufacturer’s instructions and absorbance at 490 nm (OD490) was determined for each well by Infinite^®^ M1000 Pro Microplate Reader (TECAN, Männedorf, Switzerland).

### Wound healing assay

Cells (5x10^6^) were cultured to >80% confluency in 12-well plates. After scratching a straight line through the cell layer with a sterile 200 μL pipette tip, cells were incubated with doxorubicin, CXCR4-KLA and combination treatment in an RPMI 1640 medium containing 10% FBS (w/v) for 48 h. The images of wound were taken using an EVOS^®^ cell imaging system (Life Technologies Corp., Grand Island, NY) at 10X magnification.

### Invasion assay

The *in vitro* migratory properties of cells were studied using Boyden chambers (NeuroProbe, Houston, TX) coated with collagen (Sigma, St Louis, MO). Bottom wells were filled with media (27 μL) containing 2% (w/v) of serum. Cells (5x10^4^/56 μL) were seeded on the upper compartment and then incubated at 37°C for 24 h. Cells on the upper surface of the filter were then removed using a cotton swab, leaving those attached to the lower surface stained with Diff-Quik reagents (Thermo Scientific, Waltham, MA). The numbers of invaded cells were counted under a microscope with 10X magnification (5 fields/well). A representative graph of six independent experiments was performed.

### Soft agar colony formation assay

Cells (5x10^4^) were suspended in media containing 10% (w/v) FBS and 0.35% (w/v) agar and seeded in pre-solidified media containing 0.75% (w/v) agar containing 10% (w/v) FBS in on 6-well plates. The plates were then incubated at 37°C in a humidified atmosphere of 5% CO_2_. Colonies of cells were allowed to grow for 2 weeks and any colonies larger than 0.1 mm in diameter were counted using the EVOS^®^ cell imaging system (Life Technologies Corp., Grand Island, NY) at 4X magnification.

### Xenograft

The protocol was approved by the Institutional Animal Care and Use Committee of Weill Cornell Medicine (Number: 2015–0014). All procedure was performed under isofluorane anesthesia, and all efforts were made to minimize suffering. Briefly, A2780/ADR and the freshly isolated CXCR4^High^ and CXCR4^Low^ cells (7 x 10^5^) suspended in PBS (100 μL) were injected into the flank of 6-week-old female SCID (SHO) mice (Charles River Laboratories, Wilmington, MA). The resulting tumors were measured with digital calipers and tumor volumes were calculated as follows: volume = length x width^2^ x 0.52. Samples of each tumor were fixed immediately in 10% (v/v) formaldehyde for further histology studies. Immunohistochemistry stainings were performed on the deparafiinized sections (6 μm), using antihuman CXCR4 monoclonal antibody (ab2074, Abcam, Cambridge, MA) and CD31 (1:100, ab125212, Abcam, Cambridge, MA).

For flow cytometry analysis, SKOV-3/GFP-Luc cells (2 x 10^6^) suspended in PBS (100 μL) were injected into the flank of 6-week-old female SCID (SHO) mice. When tumor reached approximately 100 mm^3^ (approximately 14 days following inoculation), mice were randomized into two groups (n = 4/group) for treatment with doxorubicin (5 mg/kg) in PBS (200 μL) or vehicle only via tail vein injections. After 72 h, tumor tissue was minced and digested with an enzyme cocktail (collagenase A, elastase, and DNase I, Roche Applied Science) in PBS at 37°C for 1 hr. The cell suspension was strained through a 40 μm cell strainer (BD Biosciences). Cell were washed with PBS three times and analyzed through flow cytometry.

### Statistics

All experiments were carried out three times. The results are presented as mean ± SD. For statistical comparisons, Graph Pad Prism 7.0 software was used to determine *p*-values using Student’s t-test and two-way ANOVA. The significance levels were set at *p<0.05, **p<0.01, and ***p<0.001.

## Results

### Chemotherapy transiently induces CXCR4^Low-High^ cell state transition in human ovarian cancer

**Fig 1:**
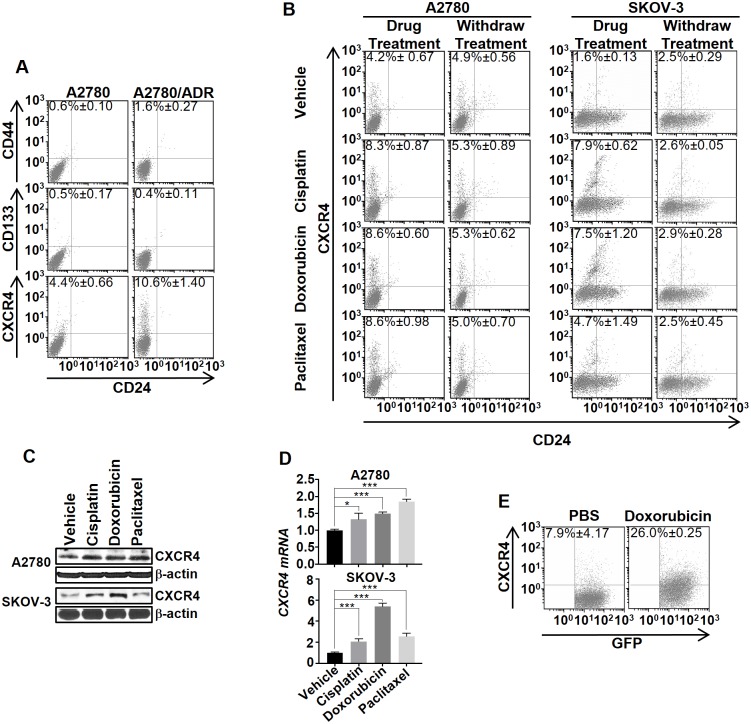
Chemotherapy transiently induced the CXCR4^High^ cell state transition in human OVC. (A) Representative FACS analysis showed that A2780/ADR displays a higher percentage of CXCR4^High^ than A2780. Cells were stained with fluorescently labeled anti-CD44, anti-CD133, or anti-CXCR4, and also with anti-CD24. The numbers in the quadrants indicate the average percentage of CXCR4^High^. (B) Chemotherapy reversibly induced CXCR4^High^ in A2780 and SKOV-3. A comparison of the changes in the cell population 72 h after treatment with suboptimal concentrations of cisplatin (1 μM), doxorubicin (10 nM), and paclitaxel (100 pM). At 72 h after withdrawing the drugs, the percentage of CXCR4^High^ returned to the basal level. (C and D) The drug-induced CXCR4^High^ was confirmed using western blot and real-time PCR analysis of the CXCR4 expression in the cell lysates. All the results represent means ± SD of three independent experiments (t-test, *p<0.05, ***p<0.001). (E) Doxorubicin induces tumoral CXCR4^+^-C. Mice (n = 3/group) were implanted with SKOV3/GFP-*Luc* (tagged with GFP and luciferase) and then treated with PBS (control) or doxorubicin (5mg/kg) via IV injection. After 72 h, the tumors were dissociated and analyzed for the percentage of CXCR4^+^-C by FACS analysis.

For many years, acquired drug resistance has been interpreted exclusively as a positive selection of distinct populations of innate drug-resistance cancer stem cells (CSCs) or tumor-initiating cells (TICs) after drug treatments [28]. Recently, Sengupta *et al* demonstrated that breast cancer cells drug resistance through an alternative route that involves a chemotherapy-induced cell state transition [29]. Here, we investigated whether such a dynamic cell state transition occurred in OVC. We first analyzed the phenotypic heterogeneity of A2780 and its doxorubicin-resistant cell lineage (A2780/ADR) by screening for the presence of live (non-fixed) cell subsets that express cell-surface CSC markers (CD44, CD133, and CXCR4) [26, 30–35]. FACS analysis showed that A2780 composed with 4.4% of CXCR4^High^/CD24^Low^ CSC population (CXCR4^High^). On the other hand, A2780/ADR, treated weekly with doxorubicin to maintain a consistent degree of drug resistance, displayed a significantly higher percentage (10.6%) of CXCR4^High^ (Fig 1A). Interestingly, we could barely detect CD44^High^/CD24^Low^ and CD133^High^/CD24^Low^ CSC populations in both A2780 and A2780/ADR. To investigate whether other chemotherapeutic treatments could induce CXCR4^High^, we incubated A2780 or SKOV-3 with suboptimal concentrations (IC_20_) of cisplatin, doxorubicin, or paclitaxel, and then performed FACS analysis of the CSC populations. In all cases, the density of CXCR4^High^ was increased significantly after 72 h (Fig 1B). The results were confirmed by the increased CXCR4’s protein and mRNA levels in the cell lysates (Fig 1C and 1D). Interestingly, the drug-induced CXCR4^Low-High^ cell transition only occurred temporarily. When the drugs were withdrawn from the cell lines, the original percentage of CXCR4^High^ was recovered after three passages (Fig 1B). We further investigated whether drug treatments enrich tumoral CXCR4^+^-C. We treated SCID mice implanted with SK-OV3 (transfected with GFP and luciferase) with doxorubicin. After 72 h, we isolated the live OVC cells (GFP+) from the tumors, and determined the percentage of CXCR4^+^-C. The CXCR4^+^-C population in the tumors of the drug-treated mice were significantly higher (7.9 vs. 26%) than in those animals received no treatment (Fig 1E), thus, suggesting that drug treatment could enrich the tumoral CXCR4^+^-C population in addition to cell cultures.

### CXCR4^High^ exhibits drug-resistant, mesenchymal, and cancer stem cell properties

**Fig 2:**
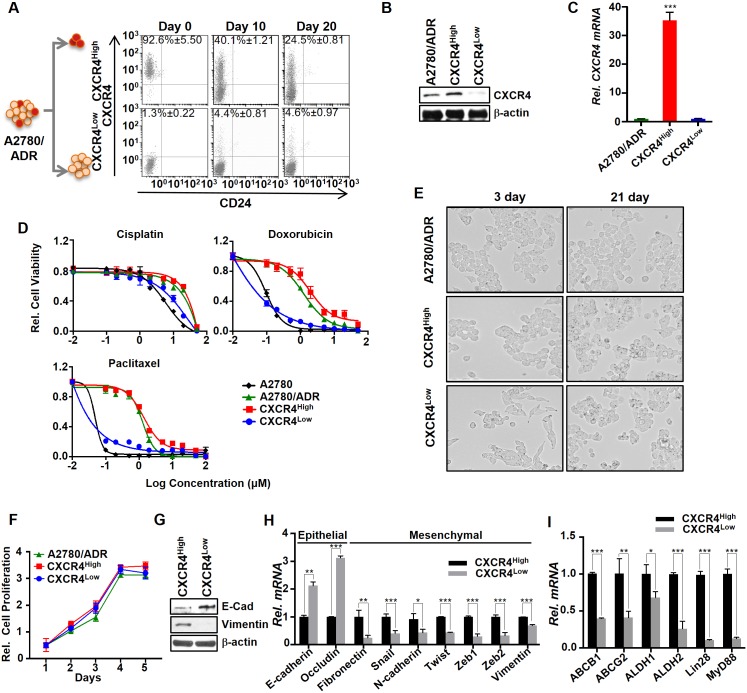
CXCR4^High^ displayed drug-resistant and mesenchymal properties. (A) FACS analysis showed that the freshly isolated CXCR4^High^ and CXCR4^Low^ eventually lost their purity over time. The cells were isolated by flow cytometry using APC-labeled CXCR4 antibody. (B and C) The CXCR4 expressions in A2780/ADR, CXCR4^High^, and CXCR4^Low^ were determined using western blot and real-time PCR analysis. Results of western blot and real-time PCR were normalized to β-actin expression and the CXCR4 mRNA expression of CXCR4 in A2780/ADR cells were set to 1. (D) Plots of relative cell viability versus different drug treatments reveal that CXCR4^High^ cells were intrinsically drug-resistant. The cell viability was determined by MTS assay. (E) Microscopic images (10x magnification) of CXCR4^High^ and CXCR4^Low^. The two freshly isolated cell lineages from A2780/ADR display significantly different morphologies. After continuous culturing for 21 days, their morphology became similar, presumably because OVC prefers to maintain an equilibrium state between the two cell populations. (F) Comparison of the cell proliferation among A2780/ADR and its isolated CXCR4^High^ and CXCR4^Low^ lineages. CXCR4^High^ exhibits the similar cell proliferation rate. (G and H) CXCR4^High^ displayed mesenchymal phenotypes, as shown by the higher and lower expressions of the epithelial and mesenchymal markers, respectively, compared to CXCR4^Low^ using western blot and real-time PCR. (I) CXCR4^High^ also displayed higher expressions of cancer stem cell markers. Results of real-time PCR was normalized to β-actin expression and the mRNA gene expression of epithelial, mesenchymal and stem cell markers in CXCR4 ^High^ cells were set to 1. All our data represent means ± SD of three independent experiments (t-test, *p<0.05, **p<0.01 and ***p<0.001).

The chemotherapy-induced CXCR4^High^ may represent a unique mechanism of acquiring drug resistance. To test this hypothesis, we first isolated CXCR4^High^ and CXCR4^Low^/CD24^Low^ (CXCR4^Low^) from A2780/ADR in high purity by flow cytometry (Fig 2A). The difference in CXCR4 expression between the two isolated cell clones was confirmed using western blot and real-time PCR (Fig 2B and 2C). Both cell clones lost purity after 20 days of continuous culturing (three passages in the absence of doxorubicin), as shown by the CXCR4^High^ clone’s dramatically reduction in the CXCR4-positive cell density (from 93% to 25%) and whereas the CXCR4^Low^ clone showed an increase of the CXCR4^High^ population (from 1% to 5%).

To investigate whether this small CXCR4^High^ played a significant role in drug resistance, we freshly isolated CXCR4^High^ and CXCR4^Low^ from A2780/ADR and compared their susceptibility to drug treatments. MTS assay showed that CXCR4^High^ was intrinsically resistant to cisplatin, doxorubicin, and paclitaxel (Fig 2D). The IC_50_ values of each drug toward CXCR4^High^ and its parent A2780/ADR were similar (Table 1). The drug-induced CXCR4^High^ contributed drug-resistance, as removing CXCR4^High^ from A2780/ADR recovered the sensitivity of the cell lines to drug treatments.

**Table 1:** Comparison of the IC_50_ values among different drugs against various OVC cell lines or cell clones.

	A2780	A2780/ADR	CXCR4^High^	CXCR4^Low^
**Cisplatin (μM)**	7.1 ± 0.4	31.8 ± 3.7	32.1 ± 4.3	8.46 ± 0.5
**Doxorubicin (μM)**	0.05 ± 0.008	1.4 ± 0.03	2.1 ± 0.1	0.05 ± 0.006
**Paclitaxel (μM)**	0.02 ± 0.002	1.3 ± 0.1	1.4 ± 0.2	0.001 ± 0.0001
**CXCR4-KLA (μM)**	10.8 ± 0.9	11.8 ± 1.0	2.6 ± 0.2	11.3 ± 0.5

Increasing studies have shown that chemotherapy induced drug resistance as well as tumorigenesis via epithelial-mesenchymal transition (EMT) in OVC [26, 36]. During our studies, we observed significant differences in the morphology between the isolated CXCR4^High^ and CXCR4^Low^ clones (Fig 2E), suggesting that chemotherapy might also induce the EMT of a small population of OVC cells. Despite the two cell clones showed a similar proliferation rate (Fig 2F), CXCR4^High^ expressed lower E-cadherin (epithelial marker) and higher Vimentin (mesenchymal marker) levels than CXCR4^Low^ (Fig 2G). The real-time PCR analysis also confirmed that CXCR4^High^ expressed lower levels of epithelial markers, including E-cadherin and Occludin, and higher levels of mesenchymal markers, including Fibronectin, Snail, N-cadherin, Twist, Zeb1, Zeb2, and Vimentin (Fig 2H). Moreover, CXCR4^High^ expressed higher level of mRNA stem cell markers including ABCB1, ABCG2, ALDH1, ALDH2, Lin28, and MyD88 (Fig 2I). Our data supported that the drug-resistant CXCR4^High^ population of A2780/ADR displayed mesenchymal CSC-like characteristics.

### CXCR4^High^ displays enhanced invasion, migration, and tumor formation properties

**Fig 3:**
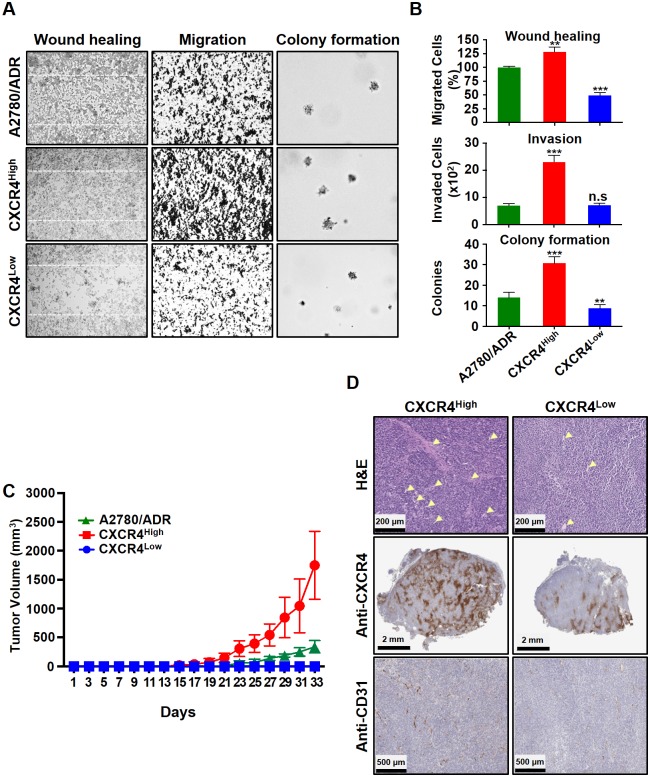
The freshly isolated CXCR4^High^ showed enhanced invasion, migration, and tumor formation properties compared to the CXCR4^Low^. (A) Microscopic images of wound healing, Boyden-chamber, and soft agar colony formation assays. (B) The results were also presented quantitatively to reflect the percentage and number of cells that invaded the wounds and migrated through the chambers, as well as the number of colonies formed. All results are presented as means ± SD of three independent experiments (t-test, **p<0.01 and ***p<0.001). (C) A comparison of the A2780/ADR, CXCR4^High^ and CXCR4^Low^ tumors’ growth rate in SCID mice (n = 5/group). The tumors were subcutaneously implanted on each side of the back of the animals. Data are presented as mean tumor volumes (mm^3^) ± SD versus time (two-way ANOVA, ***p<0.001). (D) Representative microscope images of the H&E staining and immunohistochemistry of the tumor sections show that CXCR4^High^ tumor consist of higher expressions of CXCR4 and CD31 (blood vessel). Arrows indicate the vascular core areas.

The mesenchymal nature of CXCR4^High^ may play significant roles in enhancing the tumor invasion, migration, and formation properties of OVC [26, 33]. This prompted us to investigate the migration properties of CXCR4^High^. A wound healing assay showed that the cells closed scratched wounds within 48 h, but CXCR4^Low^ was unable to complete the wound healing at this time frame (Fig 3A and 3B). We also performed Boyden chamber assay to confirm the enhanced migration of CXCR4^High^, and showed that three times more cells migrated through the chamber. When compared to CXCR4^Low^, CXCR4^High^ also produced larger and more colonies in a soft agar assay. In terms of tumor formation, the subcutaneously implanted CXCR4^High^ initiated a much faster tumor growth rate in SCID mice and after 21 days, the average size of tumors was four times larger than the implanted A2780/ADR and CXCR4^Low^ tumors (Fig 3C). Further immunohistology study showed that the excised CXCR4^High^ tumors consisted of a higher blood vessel density (Fig 3D). This was expected since the SDF-1/CXCR4 axis has been shown to participate in angiogenesis [10]. Taken together, our data strongly suggested that drug-induced CXCR4^High^ plays significant roles in tumor progression.

### CXCR4^High^ as a potential therapeutic target for OVC

**Fig 4:**
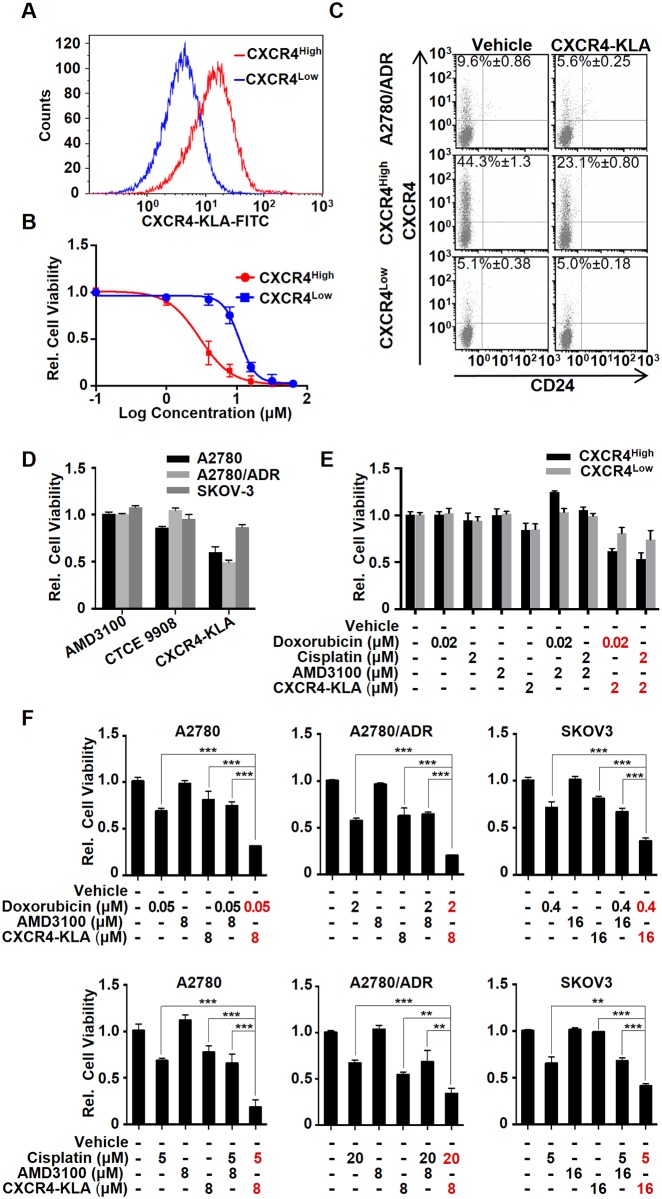
CXCR4^High^ can be a useful target for OVC treatment. **(A) CXCR4-KLA (labeled with FITC) specifically bound to CXCR4**^**High**^**.** Cells were incubated with the peptide (5 μM) in 1.5% (w/v) FBS containing medium for 2h at 37°C prior to performing FACS analysis. (B) CXCR4-KLA displays the preferential cell killing of CXCR4^High^ compared to CXCR4^Low^. MTS assays were performed on the cells 72 h after incubation with different concentrations (0–100 μM) of the peptide. (C) The specificity of CXCR4-KLA was confirmed by a reduction of the CXCR4^High^ fraction of both CXCR4^High^ and A2780/ADR after treatment with a suboptimal concentration (2 μM) of CXCR4-KLA for 72 h. (D) CXCR4-KLA is more potent than the conventional CXCR4 antagonists, including AMD3100 and CTCE9908, toward A2780, A2780/ADR, and SKOV3. Cells were incubated with the peptide (10 μM) for 72 h prior to determine the cell viability using MTS assay. (E) Using a combination of drugs (doxorubicin or cisplatin) and CXCR4-KLA (at suboptimal concentrations) showed synergistic cell-killing effects on CXCR4^High^ and CXCR4^Low^ (two way ANOVA, ***p<0.001). (F) The drug-peptide combinations also show synergistic cytotoxicity toward A2780, A2780/ADR, and SKOV-3. The drug dosages were selected according to the IC_50_ values of the drug or peptide alone against individual cell lines. All the experiments were performed in triplicate and the results were presented as means ± SD of three independent experiments (t-test, *p<0.05, **p<0.01, ***p<0.001).

The enhanced drug resistance, metastasis, and tumor formation indicated that CXCR4^High^ could be a therapeutic target for OVC treatment. Currently, there are several clinical trials of AMD 3100, CTCE-9908, and neutralizing antibody that target SDF-1/CXCR4 signaling. However, simply truncating the SDF-1/CXCR4 signaling by CXCR4 antagonists would only lead to moderate anticancer effects [21, 37]. An approach that can eliminate rather than antagonize CXCR4^High^ may increase the therapeutic efficacy. Here, we synthesized a bifunctional peptide (CXCR4-KLA) composed with a CXCR4-binding sequence (KPVSLSYRC) [38] conjugated to a mitochondrial disruption domain (KLA). Once the peptide is delivered inside a cancer cell via receptor-mediated endocytosis, the intracellular KLA domain can physically disrupt the mitochondrial membrane to induce apoptosis by releasing cytochrome *c* [27]. We first determined the specificity of the peptide. Initial FACS studies demonstrated that CXCR4-KLA selectively bound to CXCR4^High^ (Fig 4A). MTS assay showed that the peptide was 5-fold more cytotoxic toward CXCR4^High^ than CXCR4^Low^ (Fig 4B). The calculated IC_50_ values were 2.6 μM and 11.3 μM, respectively. Such a preferential cell-killing effect was further confirmed by CXCR4-KLA selectively reducing the CXCR4-positive cell density in CXCR4^High^ as well as in its parent A2780/ADR over time, as shown in FACS analysis (Fig 4C). The peptide was more potent than AMD3100 or CTCE-9908 (Fig 4D and S1 Fig). When used together with doxorubicin or cisplatin at the suboptimal concentrations (IC_20_), the drug-peptide combinations executed synergistic cell killing to both CXCR4^High^ and CXCR4^Low^ (Fig 4E).

**Fig 5:**
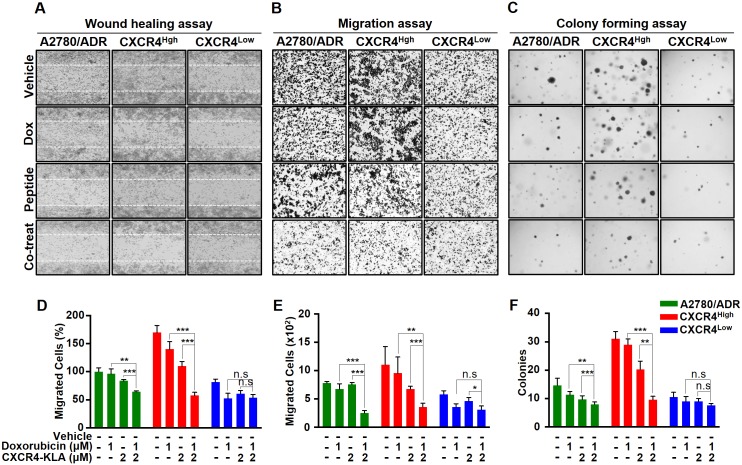
Using a combination of doxorubicin and CXCR4-KLA could synergistically inhibit invasion, migration, and colonization of OVC. (A-C) Representative images showing the wound healing, migration, and colony formation assays on A2780/ADR, CXCR4^High^, and CXCR4^Low^. The cells were treated with vehicle alone, doxorubicin alone, CXCR4-KLA alone and a combination of the drug and peptide. (D-F) The results were also presented quantitatively to reflect the percentage and number of cells that invaded the wounds and migrated through the chambers, as well as the number of colonies formed. The results expressed on the graph represent the means ± SD of three independent experiments (t-test, *p<0.05, **p<0.01, ***p<0.001).

To demonstrate the therapeutic merits of targeting CXCR4^High^, we evaluated the cytotoxicity of CXCR4-KLA alone or in combination with other cytotoxic agents toward A2780, A2780/ADR, and SKOV3. Since the potency of a drug is cell-line specific, we first determined the cytotoxicity of individual drugs or peptide toward each cell line (Table 1), and then formulated a drug-peptide combination with the ratio of the drug (doxorubicin or cisplatin) and peptide (CXCR4-KLA) selected according to the calculated IC_50_ values (e.g., Drug A: Drug B = IC_50_ of drug A: IC_50_ of drug B). We also compared the therapeutic efficacy of the drug-peptide combinations with the drug-AMD3100 combinations. Given to the AMD3100 displayed no cytotoxic effect on OVC cell lines (S2 Fig), we used the same dose of CXCR4-KLA and AMD3100 for the comparative study. As expected, drug-peptide combinations (at suboptimal IC_20_ or IC_50_ of drug and peptide dosages) were more effective than doxorubicin, cisplatin, AMD3100, or peptide alone, or any of the drug-AMD3100 combinations for eradicating A2780, A2780/ADR, and SKOV-3 (Fig 4F and S3 Fig). This is because using a cytotoxic agent and CXCR4-KLA together could simultaneously eliminate both chemo-sensitive and drug–resistant cancer cells. We also performed Chou-Talalay Plots to confirm that both the doxorubicin-CXCR4-KLA and cisplatin-CXCR4-KLA combinations were synergistic [39], with calculated combination index values of < 1 (S4 Fig). Finally, using a combination of doxorubicin and CXCR4-KLA, we further demonstrated that it could synergistically inhibit the invasion, migration, and colony formation of A2780/ADR and the isolated CXCR4^High^ (Fig 5). Overall, our results strongly suggested that CXCR4-KLA could be used together with other cytotoxic agents to achieve synergistic cell-killing and anti-metastatic effects on OVC.

## Discussion

Most OVC patients respond well to chemotherapy but quickly develop drug resistance, leading to treatment failure. Currently, there is no effective approach to prevent drug resistance in OVC. The five-year survival rate of patients is low, 30–40% [40]. To design new and more effective treatments, it is essential to understand how chemotherapy induces drug resistance. CXCR4 is a cancer stem cell (CSC) marker [26, 41]. In the present studies, we demonstrated that drug treatments caused a dynamic shift of the cell-state equilibrium in OVC cells, a transient increase in CXCR4^High^ cell population. Such a drug-induced transition event only occurred temporarily. Withdrawing the drug treatments returned to the original equilibrium status overtime. Interestingly, CXCR4^High^ and CXCR4^Low^ that were physically isolated from A2780/ADR by flow cytometry was shown to spontaneously re-establish cell-state equilibrium. After three cell passages, the isolated CXCR4^High^ population lost its purity (with 68% of the cells underwent CXCR4High⇒Low transition). On the other hand, a small percentage (4%) of the pure CXCR4^Low^ population transit to CXCR4^High^ over time. Our results were in agreement with the Markov model, which states that cancer cells can stochastically transit between states [42]. We also found the isolated CXCR4^Low^ and CXCR4^High^ were phenotypically different. CXCR4^High^ was not only more resistance to doxorubicin, paclitaxel, or cisplatin treatment but also played a dominant role in controlling acquired and possibly innate drug resistance. A selective removal of CXCR4^High^ from doxorubicin-resistant A2780/ADR could recover the sensitivity to drug treatments. The CXCR4^High^ isolated from A2780/ADR was resistance to paclitaxel and cisplatin in addition to doxorubicin, most likely because of a relative higher expression of ATP-binding cassette sub-family B member 1 (ABCB1), a common cell membrane protein that involves in acquiring multidrug resistance, compared to CXCR4^Low^. Other studies also supported ABCB1 is associated with paclitaxel-resistant A2780 cell line, leading to cross-drug resistance [43]. However, further studies are required to investigate the role of CXCR4^High^ or its associated SDF-1/CXCR4 axis to regulate ABCB1 or vice versa.

Our studies also showed that SDF-1/CXCR4 axis is involved in EMT of various cancers including lung, pancreatic, hepatic, and ovarian cancers [44–46]. Here, it was not a surprise to find CXCR4^High^ displaying mesenchymal-like characteristics, a lower epithelial marker and higher mesenchymal marker levels with enhanced invasion, migration and tumor formation properties compared to CXCR4^Low^. Given to the dominant role of CXCR4^High^ in acquiring drug resistance and metastatic potential, we suggested that a means to truncate CXCR4^High^ would improve chemotherapeutic treatment outcomes. We designed and synthesized CXCR4-KLA to investigate the therapeutic merits of targeting CXCR4^High^. Unlike the conventional antagonists that only block the activities of SDF-1/CXCR4 axis, CXCR4-KLA can eliminate CXCR4^High^. This peptide was shown to be more potent than AMD3100 and CTCE-9908. When used in combination with other cytotoxic agents including doxorubicin and cisplatin, it displays synergistic cytotoxicity and the inhibition of cell invasion, migration, and colony formation in OVC. Currently, there are very few reports of using CXCR4 antagonists in combination therapy of OVC [22, 37]. Overall, we demonstrated that therapeutic strategies designed to eradicate rather than antagonize CXCR4^High^/CD24^Low^ might offer far-reaching potential as a supportive therapy. One advantage of CXCR4-KLA is that it can be easily modified to target other cancer cells [47]. Given to a tumor consists of heterogeneous cell populations, we could easily replace the binding domain of CXCR4-KLA with other ligands to target other CSCs that express different cell-surface biomarkers such as CD133, CD44, CD117, and MyD88 of OVC in the future [48–51]. This can be a useful approach to study the functional roles and targeting benefits of a particular or multiple CSC populations.

## Supporting information

S1 Fig MTS assay. AMD3100 displayed no cytotoxic effect on the tested OVC cell lines.
(TIF)

S2 Fig A comparison of the cytotoxicity among AMD3100, CTCE9908, and CXCR4-KLA towards different OVC cell lines. CXCR4-KLA is more cytotoxic presumably the peptide induced the apoptosis of CXCR4^High^ rather than antagonize the CSCs.
(TIF)

S3 Fig The drug-peptide combinations show synergistic cytotoxicity towards A2780, A2780/ADR, and SKOV-3. The drug dosages were selected according to the IC_20_ values of the drug or peptide alone against individual cell lines. All the experiments were performed in triplicate and the results were presented as means ± SD of three independent experiments (t-test, *p<0.05, **p<0.01, ***p<0.001).
(TIF)

S4 Fig Chou-Talalay Plot of different drug combinations tested on various OVC cell lines. The data show that the calculated combination index (CI values) of doxorubicin-CXCR4-KLA and cisplatin-CXCR4-KLA combinations. Both drug combinations show synergistic cell killing (CI<1) at doses that kill more than 60% of the cells (where Fraction affected = 1-relative cell viability). Methods: Different concentrations of doxorubicin, cisplatin, or peptide alone, or the drug-peptide combinations (applied with drug ratios based on the IC_50_ values of drugs or peptide alone towards individual cell lines) were added to cancer cells pre-seeded in a 96-well plate (2000 cells) for incubation. After 72 h, the cell viability was determined by MTS assay. The data were analyzed using the CalcuSyn software as previously described [39]. Data are averages of triplicate determinations ± SEM.
(TIF)

S1 Table A list of the primers used for RT-PCR analysis. (DOCX)
