# Supplementary figures and images for: Chemotherapy induces adaptive drug resistance and metastatic potentials via phenotypic CXCR4-expressing cell state transition in ovarian cancer
Source: PLoS One. 2017 Feb 14;12(2):e0171044. doi: 10.1371/journal.pone.0171044 (PMC5308810; doi:10.1371/journal.pone.0171044)

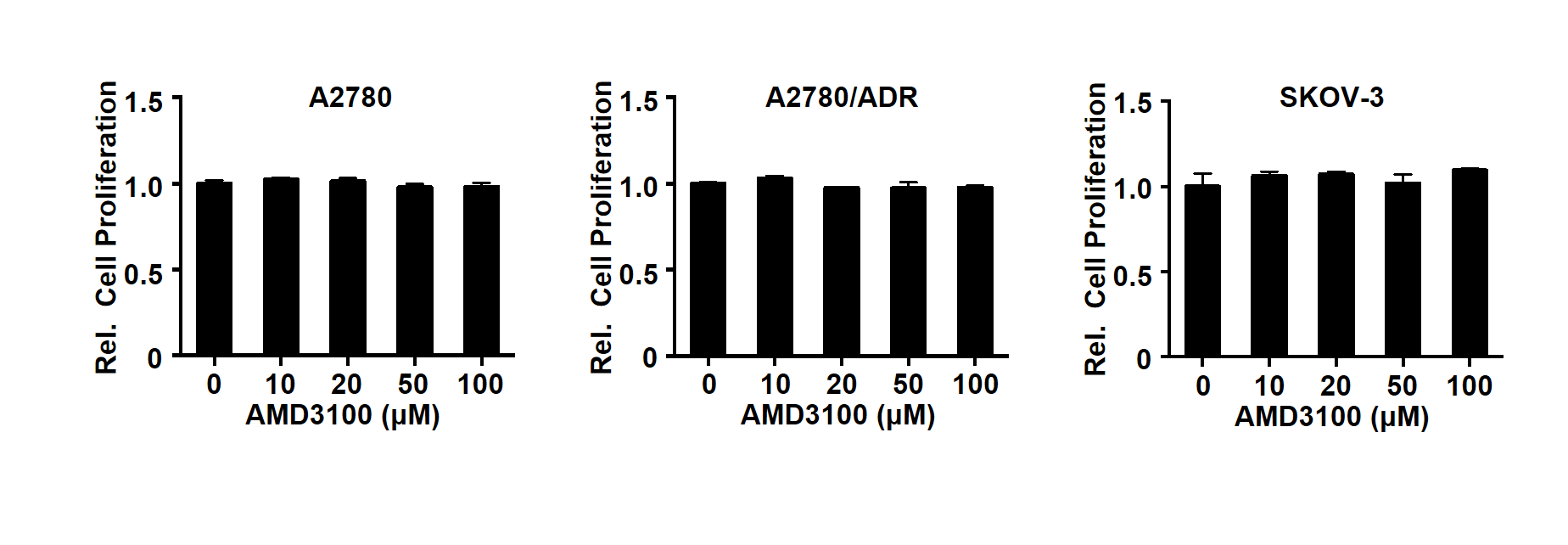

Supplement: S1 Fig — AMD3100 displayed no cytotoxic effect on the tested OVC cell lines. (TIF) [file pone.0171044.s001.tif]

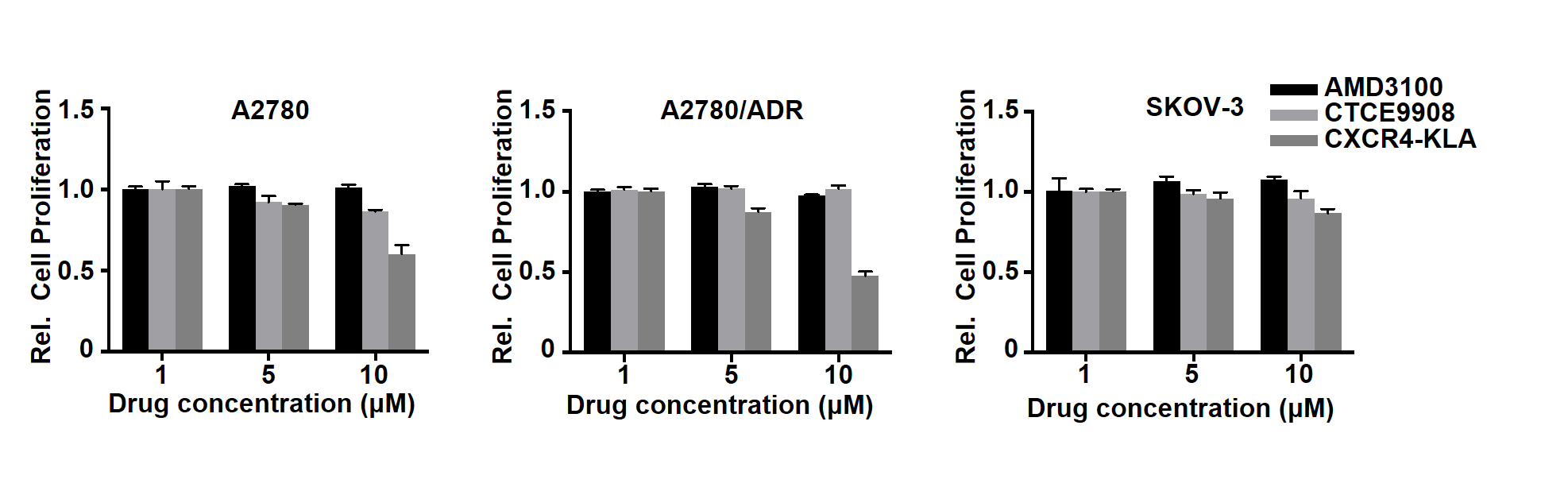

Supplement: S2 Fig — CXCR4-KLA is more cytotoxic presumably the peptide induced the apoptosis of CXCR4High rather than antagonize the CSCs. (TIF) [file pone.0171044.s002.tif]

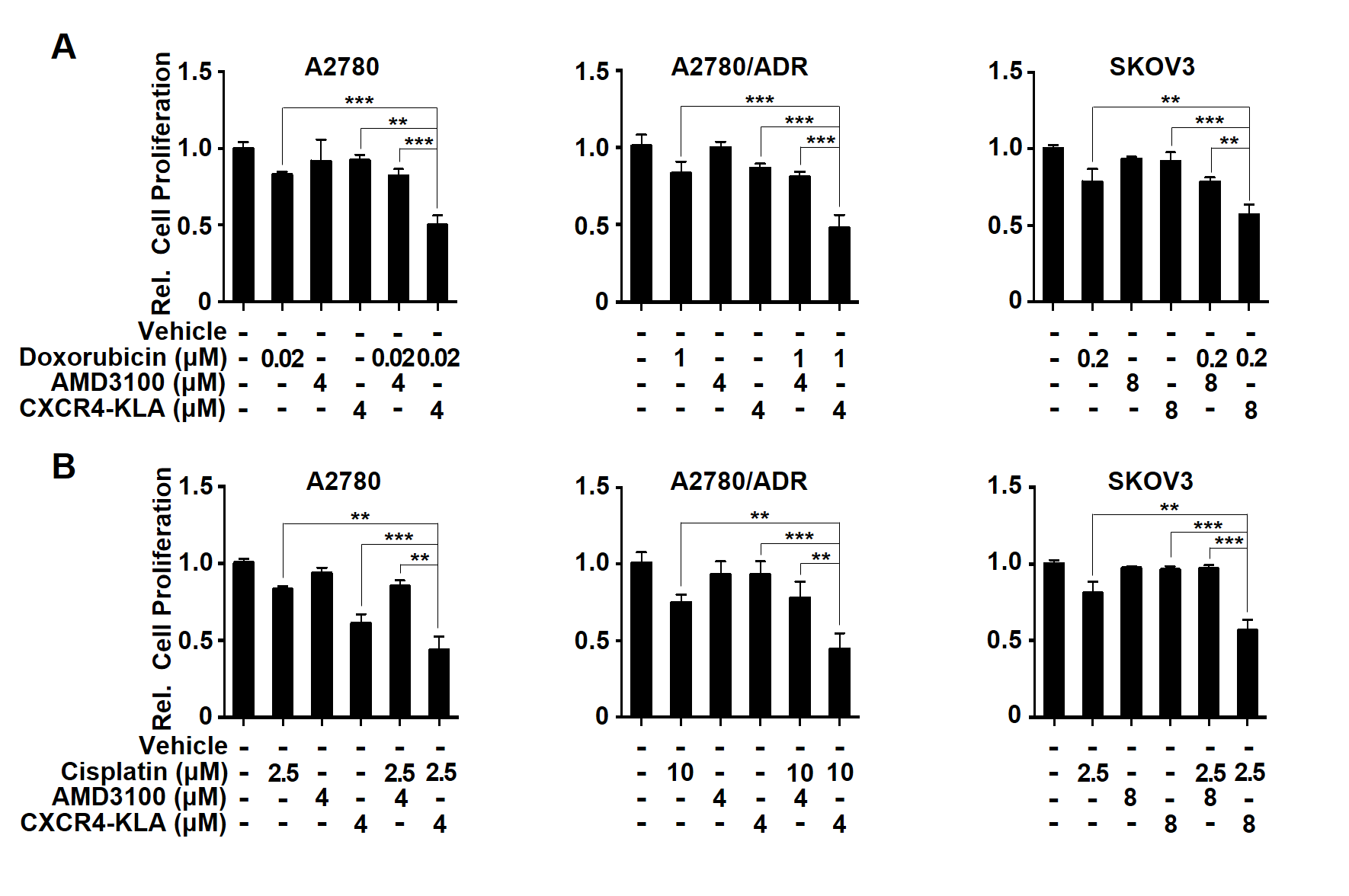

Supplement: S3 Fig — The drug dosages were selected according to the IC20 values of the drug or peptide alone against individual cell lines. All the experiments were performed in triplicate and the results were presented as means ± SD of three independent experiments (t-test, *p<0.05, **p<0.01, ***p<0.001). (TIF) [file pone.0171044.s003.tif]

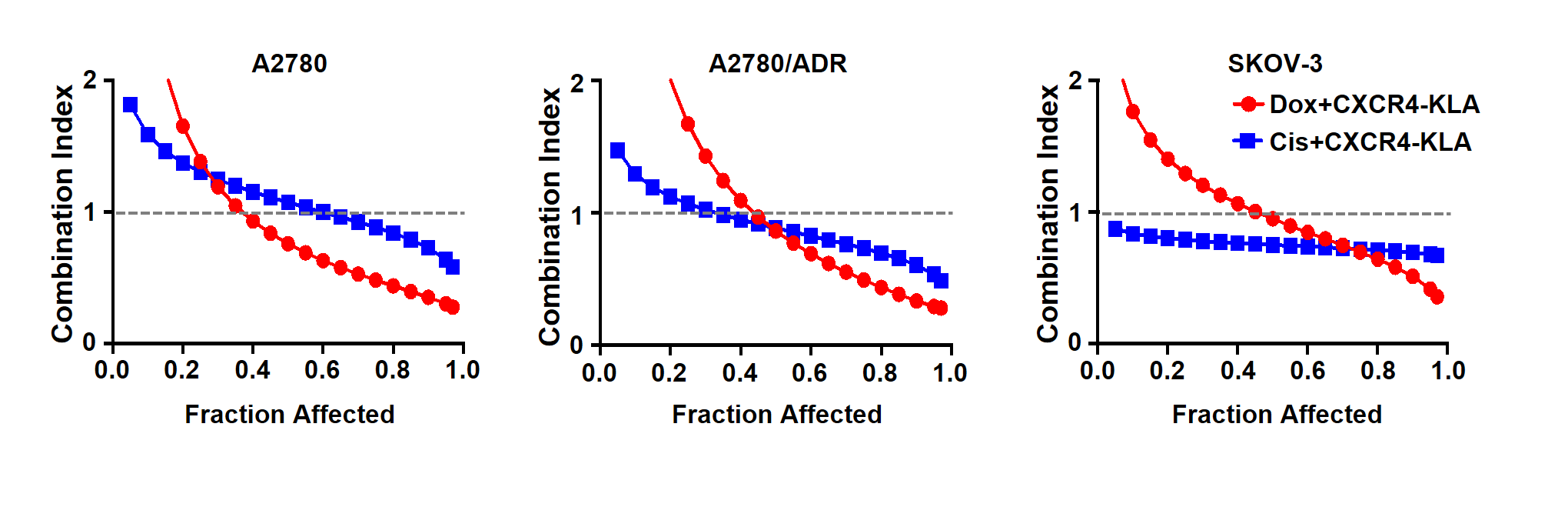

Supplement: S4 Fig — The data show that the calculated combination index (CI values) of doxorubicin-CXCR4-KLA and cisplatin-CXCR4-KLA combinations. Both drug combinations show synergistic cell killing (CI<1) at doses that kill more than 60% of the cells (where Fraction affected = 1-relative cell viability). Methods: Different concentrations of doxorubicin, cisplatin, or peptide alone, or the drug-peptide combinations (applied with drug ratios based on the IC50 values of drugs or peptide alone towards individual cell lines) were added to cancer cells pre-seeded in a 96-well plate (2000 cells) for incubation. After 72 h, the cell viability was determined by MTS assay. The data were analyzed using the CalcuSyn software as previously described [39]. Data are averages of triplicate determinations ± SEM. (TIF) [file pone.0171044.s004.tif]
