# Supplementary material for: Chemotherapy induces adaptive drug resistance and metastatic potentials via phenotypic CXCR4-expressing cell state transition in ovarian cancer
Source: PLoS One. 2017 Feb 14;12(2):e0171044. doi: 10.1371/journal.pone.0171044 (PMC5308810; doi:10.1371/journal.pone.0171044)
Supplement: S1 Table — (DOCX) [file pone.0171044.s005.docx]

**Table S1: A list of the primers used for RT-PCR analysis.**

| **Gene** | **FoMyD88rward** | **Reverse** |
| --- | --- | --- |
| **CXCR4** | ACACCGATGGTGAGGGTACACAGG | GTTCTCAAACTCACACCCTTGC |
| **b-actin** | GAGCGCGGCTACAGCTT | TCCTTAATGTCACGCACGATTT |
| **E-cadherin** | ACACCGATGGTGAGGGTACACAGG | GCCGCCACACACACAGCATAGTCTC |
| **Occludin** | TGCTAAGGCAGTTTTGGCTAAGTCT | AAAAACAGTGGTGGGGAACGTG |
| **Fibronectin** | GCCAATAAGCAAACGATTCTG | TTTGGCTGGATCACTTTCAAG |
| **N-cadherin** | AAGCCAGGGACAGATCAGC | CCACACTCTGTGCATTTGAAC |
| **Twist** | GCCGGAGACCTAGATGTCATT | CACGCCCTGTTTCTTTGAAT |
| **Snail** | ACTGGTGAGAAGCCATTCTCCT | CTGGCACTGGTATCTCTTCACA |
| **Zeb1** | GCCAATAAGCAAACGATTCTG | TTTGGCTGGATCACTTTCAAG |
| **Zeb2** | AAGCCAGGGACAGATCAGC | CCACACTCTGTGCATTTGAAC |
| **Vimentin** | GAACGCCAGATGCGTGAAATG | CCAGAGGGAGTGAATCCAGATTA |
| **ABCB1** | GCCTGGCAGCTGGAAGACAAATAC | ATGGCCAAAATCACAAGGGTTAGC |
| **ABCG2** | TGAGCCTTTGGTTAAGACCG | TGGTGTTTCCTTGTGACACTG |
| **ALDH1** | TGTTAGCTGATGCCGACTTG | TTCTTAGCCCGCTCAACACT |
| **ALDH2** | TTCAACCAGGGCCAGTGCTGCTGT | CCCCTCTTGCTTCCCCGTGTTGAT |
| **Lin28** | AGTGGCCTGGATAGGGAAGT | CTTGGCTCCATGAATCTGGT |
| **MyD88** | GCACATGGGCACATACAGAC | TAGCTGTTCCTGGGAGCTGT |
